# Preclinical relevance of dosing time for the therapeutic index of gemcitabine–cisplatin

**DOI:** 10.1038/sj.bjc.6602564

**Published:** 2005-04-19

**Authors:** X M Li, K Tanaka, J Sun, E Filipski, L Kayitalire, C Focan, F Lévi

**Affiliations:** 1 INSERM E 354 ‘Chronothérapeutique des Cancers’ and Université Paris XI, Hôpital Paul Brousse, 14-16 Avenue Paul Vaillant Couturier, Villejuif 94800, France; 2 Department of Surgery, Yokohama City University Hospital, Yokohama 236-0004, Japan; 3 Department of Applied Technology, Cancer Center, Sun Yat-sen University, Guangzhou 510060, China; 4 Eli Lilly and Co., Indianapolis, Indiana 46225, USA; 5 Chronotherapy Group of European Organization for Research and Treatment of Cancer, Les Cliniques Saint Joseph, 4000 Liège, Belgium

**Keywords:** chronopharmacology, gemcitabine, cisplatin, Glasgow osteosarcoma, mice

## Abstract

The relevance of gemcitabine timing for chronotherapeutic optimisation was investigated. Healthy mice received multiple doses of gemcitabine (120, 160 or 200 mg kg^−1^ injection (inj)^−1^) at one of six circadian times (3, 7, 11, 15, 19 or 23 h after light onset – HALO) on days 1, 4, 7 and 10 or a single dose of gemcitabine (400 mg kg^−1^) at 11 or 23 HALO±cisplatin (5 mg kg^−1^ at 1 min, 4 or 8 h later). Mice bearing Glasgow osteosarcoma received multiple doses of gemcitabine (200 mg kg^−1^ inj^−1^) at 11 or 23 HALO±cisplatin (5 mg kg^−1^ inj^−1^ at 1 min or 4 h later) on days of 10, 13, 16 and 19 following tumour inoculation. A circadian rhythm in body weight loss was statistically validated, with 1030 HALO corresponding to the least toxic time (95% CL, 0800 to 1300). Gemcitabine dosing produced least body weight loss and least neutropenia after injection at 11 *vs* 23 HALO, whether the drug was given alone or with cisplatin (*P*=0.001). Gemcitabine–cisplatin tolerability was improved by dosing gemcitabine at 11 HALO and CDDP at 15 HALO (*P*<0.001). The administration of this schedule to tumour-bearing mice increased median survival three-fold as compared to treatments where both drugs were given simultaneously at 11 or 23 HALO (*P*=0.02). The optimal schedule would correspond to the delivery of gemcitabine upon awakening and cisplatin near mid-activity in cancer patients.

Gemcitabine (2′,2′-difluoro-2′-deoxycytidine) is a deoxycytidine analogue that exerts its antitumour activity via multiple mechanisms of action. Gemcitabine undergoes intracellular phosphorylation to the active metabolites gemcitabine diphosphate and gemcitabine triphosphate, leading to inhibition of ribonucleotide reductase and incorporation of gemcitabine triphosphate into DNA and RNA (Xu and Plunkett, 1992; Ruiz van Haperen *et al*, 1993). It is active against non-small-cell lung cancer, pancreatic cancer, breast cancer and ovarian cancer (Abratt *et al*, 1994; Lund *et al*, 1994; Carmichael *et al*, 1995, 1996). A review of gemcitabine safety profile establishes this drug as a relatively safe antimetabolite, with adverse events that generally are manageable and reversible, rarely leading to discontinuation of the drug. Of 979 patients included in an overall safety database, less than 1% discontinued treatment due to haematological, gastrointestinal, hepatic, or other symptomatic events such as fever, oedema, rash or alopecia (Data on file. Eli Lilly & Co., Indianapolis, IN, USA). However, reports of severe and sometimes fatal lung or capillary toxicities have been observed sporadically, while myelosuppression is the dose-limiting toxicity (Kaye, 1994; De Pas *et al*, 2001; Rosado *et al*, 2002).

Combination chemotherapy, particularly cisplatin (CDDP)-based regimes, results in higher response rates as compared to single-agent chemotherapy. Preclinical studies have shown additive and synergistic effects of gemcitabine and CDDP in combination. Gemcitabine increases the formation of DNA-platinum adducts, while CDDP increases the incorporation of gemcitabine into DNA-platinum adducts (van Moorsel *et al*, 1999a; Peters *et al*, 2000). This combination has shown significant activity in a number of tumour types and has become a standard regime in advanced lung cancer (Rinaldi *et al*, 2000; Sandler *et al*, 2000). However, increased toxicities resulted from combining gemcitabine with CDDP or carboplatin have been reported (Rinaldi *et al*, 2000; Sandler *et al*, 2000; Thomas *et al*, 2002).

Changing the timing of administration along the 24-h time scale can profoundly modify the extent of dose-limiting toxicities of anticancer agents (Lévi, 1997, 2001). The adaptation of several cancer chemotherapy regimens to circadian rhythms improved their safety as well as their antitumour activity in patients (Hrushesky, 1985; Lévi *et al*, 1997).

We investigated the relationship between the circadian rhythm in the tolerability and the anticancer efficacy of gemcitabine in mice, as a prerequisite for the development of chronotherapy schedules with this drug in human cancer. Gemcitabine was first given as a single agent, either as a single dose or according to a repeat dosing schedule, previously shown to achieve good tolerability and efficacy in mouse tumour models (Braakhuis *et al*, 1995). The effect of CDDP addition on gemcitabine was further investigated as a function of dosing time.

## MATERIAL AND METHODS

### Animals and synchronisation

All experiments were carried out in accordance with the guidelines for the welfare of animals in experimental neoplasia approved by the UKCCCR (1998).

Male B6D2F_1_ mice bred by Charles River (l'Arbresle, France) were 6 weeks of age upon arrival. They were housed two or three per cage with food and water provided *ad libitum*. All mice were synchronised with an alternation of 12 h of light and 12 h of darkness for 3 weeks in an autonomous chronobiological animal facility (Jouan, Saint-Herblain, France). The facility has six soundproof, temperature-controlled compartments, each having its own programmable lighting regimen. Each compartment was constantly provided with filtered air delivered at an adjusted rate. Synchronisation was checked by the assessment of a normal circadian variation in the rectal temperature measured before treatment initiation.

### Tumour model

Glasgow osteosarcoma (GOS) was provided by the Research Centre of Aventis Pharma (Vitry sur Seine, France). The tumour was maintained in C57BL/6 female mice over 6 weeks of age and passaged every 2 weeks as bilateral subcutaneous implants in donor female C57BL/6 mice until the lower tumour weight reached 700 mg.

Donor mice were killed, their tumour were removed, placed into Hank's balanced salt solution and dissected into fragments measuring approximately 3 × 3 mm^2^ using a grill scaled to these values. Recipient experimental mice were transplanted with one tumour fragment in each flank, using a trocar. Bilateral implants were used to ensure a more-uniform tumour burden per mouse. Since the circadian time of tumour inoculation did not influence tumour growth and survival of GOS-bearing mice (Granda *et al*, 2002), tumour was inoculated to all mice between 1000 and 1200 for chronoefficacy study.

The day of tumour transplantation was considered as day 0. Tumours were measured every 2–3 days (length and width) with a sliding caliper by the same investigator. Tumour weight was calculated from caliper measurements using the following formula: tumour weight (mg)=(length × width^2^)/2. Animals with tumour reaching 10% of initial mouse body weight along the course of the study were killed by cervical dislocation for ethical reasons and considered as dead from tumour progression on this day. The number of days to reach this end point was used as a survival time estimate.

### Drugs

Gemcitabine were kindly provided by Eli Lilly (Indianapolis, IN, USA). CDDP was purchased from Eli Lilly. Both were diluted in 0.9% NaCl on each study day and injected intravenously (10 ml kg^−1^ of body weight) into the right retro-orbital venous sinus. Control mice received 0.9% NaCl.

### Study design

#### Chronotolerance

Two experiments (Exp) were performed in a total of 222 mice.

In Exp 1, gemcitabine (120, 160 or 200 mg kg^−1^ injection^−1^ – inj) was given at one of six circadian times, expressed in hours after light onset (HALO). Three, 7 and 11 HALO are located during the light span, when mice are usually at rest, while 15, 19 and 23 HALO correspond to the dark span, when mice are usually active. The drug was injected every 3 days for 10 days to 144 mice. Lethal toxicity and body weight were monitored daily for 14 days.

In Exp 2, gemcitabine (400 mg kg^−1^) was administered at 11 or 23 HALO, as a single agent or combined with CDDP (5 mg kg^−1^) to 78 mice. CDDP was given 1 min, 4 h or 8 h after gemcitabine. The main end points were survival, body weight and circulating neutrophil counts. For each mouse, blood (0.3 ml) was collected at 5 HALO, a time corresponding to the physiologic acrophase of circulating leukocytes, lymphocytes and neutrophils (Swoyer *et al* 1978) over the 6 days following drug dosing. Neutrophil count was determined with Cell-Dyn (Abbott Diagnostics, Rungis, France).

### Chronoefficacy

In Exp 3, single agent gemcitabine (200 mg kg^−1^ inj^−1^) or gemcitabine–cisplatin (200 and 5 mg kg^−1^ inj^−1^, respectively) were given to mice with advanced GOS (600–800 mg) at 11 or 23 HALO. CDDP was given 1 min or 4 h after gemcitabine. The treatment was delivered 10, 13, 16 and 19 days after tumour inoculation. Tumour growth and survival were monitored for 90 days.

### Statistical analysis

Mean±1 s.e.m. were calculated for each variable. Differences between groups were analysed with one- or two-way analysis of variance (ANOVA). Survival curves were drawn according to Kaplan–Meier, and differences in survival were tested using the log-rank method. The time series data of body weight change were further analysed by cosinor for 24 periodicity. This method computes the mesor (midline estimating statistic of rhythm or rhythm-adjusted mean), amplitude (half the difference between maximum and minimum in fitted cosine function) and acrophase (time of maximum in fitted cosine function). The 95% confidence limits of these parameters were calculated. A value of *P*<0.05 was required for statistical significance.

## RESULTS

### Chronotolerance

#### Multiple doses of single agent gemcitabine

**Figure 1:**
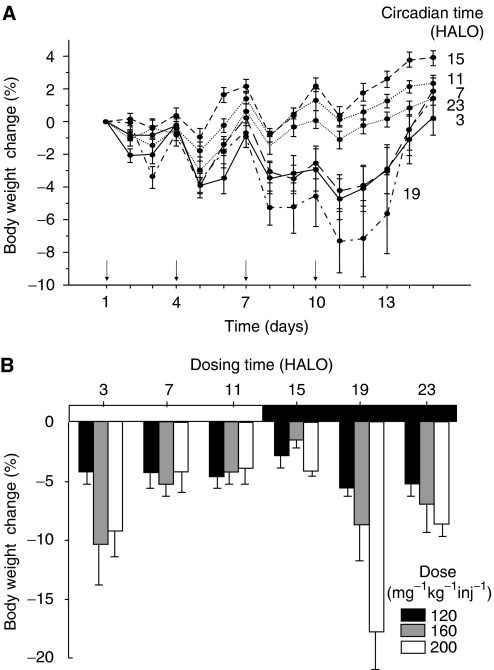
Body weight change relative to pretreatment value (mean±s.e.m.) in healthy B6D2F_1_ mice receiving gemcitabine (**↓**) on days 1, 4, 7 and 10 at one of six circadian times. Circadian times are expressed in hours after light onset (HALO). (**A**) Body weight change as a function of gemcitabine timing over 2 weeks following treatment onset at one of three dose levels (120, 160 or 200 mg kg^−1^ inj^−1^) (*P* from two-way ANOVA<0.001 for effects of dose and circadian time). (**B**) Body weight change at nadir as a function of dose and dosing time.

In Exp 1, three of 48 (6%) mice died from toxicity 11 or 12 days after treatment onset with 200 mg kg^−1^ inj^−1^, as compared to none of the mice receiving any lower dose. Lethal toxicity was only encountered following gemcitabine administration at 19 HALO when it was 37.5% (three out of eight), as compared to 0% in groups receiving the same dose at any other time. Mean maximum body weight loss was reached 1 day after the fourth dose and ranged from 1.1±0.4% (120 mg kg^−1^ inj^−1^) to 2.7±0.8% (160 mg kg^−1^ inj^−1^) and 4.9±1.1% (200 mg kg^−1^ inj^−1^) (ANOVA *P*=0.008). Irrespective of dose level, mean body weight loss varied from 7.4±1.9% in the mice treated at 19 HALO, as compared to 0.1±0.4% in those receiving gemcitabine at 11 HALO (*P*<0.001) (Figure 1A). Furthermore, the dose–toxicity relation was much steeper if gemcitabine was given at 19, 23 or 3 HALO as compared to 7, 11 or 15 HALO (*P*<0.001 for effects of dose and time) (Figure 1B). A circadian rhythm in body weight change was further statistically validated by cosinor analysis, with an acrophase corresponding to the least toxic time which was located at 1030 HALO (95% CL, 800 to 1300) (*P*=0.005).

### Single dose of gemcitabine±CDDP

#### Body weight loss

No lethal toxicity was encountered in Exp 2. The average maximum weight loss following a single dose of 400 mg kg^−1^ of gemcitabine was reached 4 days after treatment. It was 4.1±1.4% with gemcitabine alone and 7.4±0.6% with gemcitabine+CDDP. Body weight loss was significantly less in the mice given gemcitabine at 11 HALO as compared to 23 HALO, whether gemcitabine was given alone or with CDDP, irrespective of interval between both drugs (Table 1).

#### Haematological toxicity

Neutropenia reached a nadir 3 days following dosing, with full recovery 3 days later. The neutropenia nadir was further decreased with CDDP addition. Interval between gemcitabine and CDDP did not significantly influence neutropenia (*P* from ANOVA=0.55). However, neutropenia was more severe following injection at 23 HALO as compared to 11 HALO, whether gemcitabine was given alone or with CDDP (*P*⩽0.001) (Table 1).

### Effect of schedule of gemcitabine–CDDP combination

**Figure 2:**
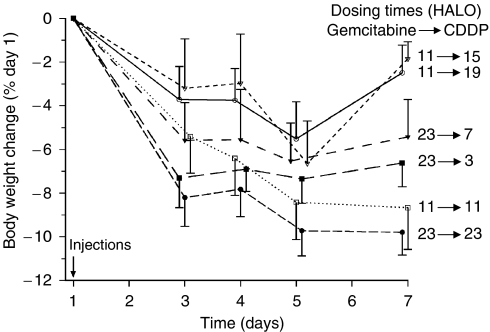
Body weight change relative to pretreatment value (mean±s.e.m.) in healthy mice over the week following a single treatment with gemcitabine (400 mg kg^−1^) and CDDP (5 mg kg^−1^). Gemcitabine was given at 11 or 23 HALO and CDDP was administered 1 min, 4 h or 8 h after eight gemcitabine timing (*P* from two-way ANOVA=0.001 for circadian time effect and <0.001 for interval effect).

Recovery from weight loss following single dose of gemcitabine combined with CDDP was influenced by gemcitabine dosing time and the interval between both drugs. Combining both effects led to contrast the worst schedule, consisting in the delivery of both agents at 23 HALO from two ‘best’ schedules, consisting in the administration of gemcitabine at 11 HALO and CDDP at 15 or 19 HALO (Figure 2). These findings were validated by two-way ANOVA, which indicated statistically significant differences as a function of both gemcitabine timing (*P*=0.001) and interval between the drugs (*P*<0.001).

#### Chronoefficacy of gemcitabine–CDDP

No toxic death was recorded after administration of gemcitabine alone in Exp 3. However, gemcitabine–CDDP induced 19 toxic deaths in 40 mice (47.5%), 3–13 days following treatment completion. No mortality was found in the mice given gemcitabine at 11 HALO then CDDP at 15 HALO. Conversely, the rates of toxic death were 50% in mice receiving gemcitabine at 23 HALO then CDDP at 4 HALO and 70% in those receiving gemcitabine and CDDP concurrently at 11 or 23 HALO (Fisher's exact test *P*=0.002).

**Figure 3:**
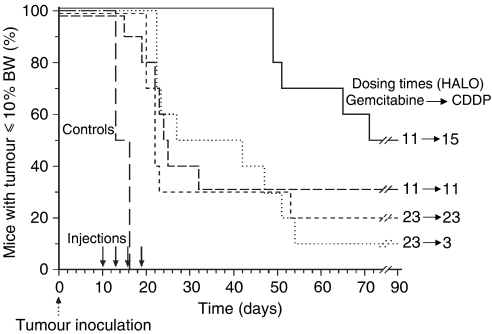
Survival curves of GOS-bearing mice receiving gemcitabine (200 mg kg^−1^ inj^−1^) combined with CDDP (5 mg kg^−1^ inj^−1^) 10, 13, 16 and 19 after tumour inoculation. Gemcitabine was given at 11 or 23 HALO and CDDP was injected 1 min or 4 h after gemcitabine timing (*P* from log rank=0.02). BW: body weight.

The overall survival estimate of GOS-bearing mice was prolonged from a median of 13 days in controls to 37 days in mice given gemcitabine alone or 42 days in those receiving gemcitabine–CDDP, irrespective of dosing time and interval between both drugs (log rank *P*<0.001). No significant difference was found between the groups given single agent gemcitabine at 11 or 23 HALO. Gemcitabine–CDDP combination proved of benefit as compared to single agent gemcitabine. Such efficacy varied significantly as a function of gemcitabine dosing time and combination schedule. Median survival time estimate was prolonged from 23 days in mice receiving gemcitabine at 23 HALO to 49 days in those receiving gemcitabine at 11 HALO, irrespective of interval between gemcitabine and CDDP (log rank *P*=0.04). Median survival time estimate ranged from 22 or 24 days following the concurrent delivery of gemcitabine and CDDP at 23 or 11 HALO up to 68 days in the mice given gemcitabine at 11 HALO then CDDP at 15 HALO (log rank *P*=0.02) (Figure 3).

## DISCUSSION

Although the schedule dependency of gemcitabine has not been fully investigated in humans, the weekly interval of gemcitabine has been largely recommended in cancer patients. Twice weekly administration of gemcitabine to patients showed a higher incidence of non haematological toxicity, for example, flu-like symptoms and rash and a lower dose intensity overall (Lund *et al*, 1993). On the basis of the schedule-dependent antitumour effect of gemcitabine, the optimal time interval between bolus injections was reported to be 3 days in mice (Lund *et al*, 1993; Kaye, 1994). The toxicity for normal tissues was also reported to be reduced by such 3-day interval between consecutive injections (Braakhuis *et al*, 1995). The every 3 days × 4 schedule, 120 mg kg^−1^ inj^−1^ on days 1, 4, 7 and 10, produced a reversible weight loss of 5–15% in tumour-bearing mice (Lund *et al*, 1993; Braakhuis *et al*, 1995). In the present study, healthy mice receiving 120 mg kg^−1^ inj^−1^ hardly lost any weight. Maximum body weight loss was less than 5% following the administration of 160 or 200 mg kg^−1^ inj^−1^ × 4. However, the tolerability of gemcitabine was several fold better following dosing during the late light to early dark span (7–15 HALO) as compared to late darkness to early light (19–3 HALO). Mean body weight loss was three-fold as large in the mice treated with 200 mg kg^−1^ inj^−1^ at 19 HALO as compared to those receiving gemcitabine between 7 and 15 HALO. According to cosinor analysis, least gemcitabine toxicity corresponded to an administration of this drug at 1030 HALO, in the late resting span of mice. Subsequent experiments have compared overall and haematological toxicities of gemcitabine as a function of whether the drug was given at 11 HALO, close to the time of best tolerability, or at 23 HALO, close to the time of worst tolerability. These experiments have clearly confirmed that gemcitabine administration at 11 HALO produced least body weight loss as compared to treatment at 23 HALO, whether gemcitabine was given as a single agent or combined with CDDP. Furthermore, the optimal interval between gemcitabine and CDDP was 4 or 8 h, in accordance with prior reports (van Moorsel *et al*, 1999b, 2000). Our study has thus identified an optimal combination schedule where gemcitabine is given at 11 HALO and CDDP at 15 or 19 HALO, that is, the respective times of least toxicity for each drug alone. The haematological toxicity of gemcitabine was primarily exerted upon neutrophil count. It was enhanced with CDDP addition and reduced in the mice given gemcitabine at 11 HALO as compared to 23 HALO. This indicated that neutropenia was one of the mechanisms of the dosing time dependent toxicity of gemcitabine±CDDP. Yet, the sparning of intestinal and lung toxicity with gemcitabine timing could also contribute to circadian optimisation. Thus, both tissues represent toxicity targets for this drug and display circadian rhythms in cellular proliferation and metabolism (Scheving and Jin, 1999; Hastings *et al*, 2003).

Gemcitabine is an S-phase specific agent (Tolis *et al*, 1999) and elicits apoptosis (Tolis *et al*, 1999; Giovannetti *et al*, 2005). This process can be prevented by BCL-2 expression and favoured by BAX expression (Hao *et al*, 2003). Indeed, the proportion of S-phase cells in the bone marrow of the mouse, we used here was highest in the second half of darkness, when locomotor activity was highest (Tampellini *et al*, 1998; Filipski *et al*, 2004). As this circadian stage, BAX expression was highest and BCL-2 expression was low (Granda *et al*, 2005). The rhythms in S-phase distribution and BCL-2/BAX expression are consistent with a better tolerability of gemcitabine during the light span. Conversely, circadian changes in S-phase and BCL-2 are usually markedly altered in experimental tumours (Granda and Lévi, 2002; Granda *et al*, 2005). The deregulation of G1–S checkpoint control by the circadian clock can relate to altered clock gene expression patterns in the tumour, as it was found for GOS (Filipski *et al*, 2004, 2005). Conversely, the circadian control of G2–M checkpoint appears to be maintained in most experimental malignances, a finding consistent with the circadian dependency of CDDP or oxaliplatin antitumour activity (Granda and Lévi, 2002; Granda *et al*, 2002, 2005).

The higher dose level of gemcitabine (200 mg kg^−1^ inj^−1^) was used here to investigate the circadian dependency of antitumour efficacy, since no toxic death was uncounted and mean body weight loss was less 10% following dosing at 11 or 23 HALO. In mice bearing GOS at advanced stage of growth, we first confirmed the better efficacy of gemcitabine–CDDP over gemcitabine alone. We further found that the delivery of gemcitabine at 11 HALO and CDDP at 15 HALO increased median survival three-fold, as compared with other schedules. The results support that a synergistic activity of these drugs requires their administration near their respective ‘best’ circadian times. They are in line with the coincidence between the time of best efficacy and that of best tolerability, which was recently shown for single agent doxorubicin, docetaxel or vinorelbine and for docetaxel–doxorubicin or irinotecan–oxaliplatin combination (Tampellini *et al*, 1998; Filipski *et al*, 1999; Granda *et al*, 2001, 2002). Although we have not explored the relevance of this best treatment schedule in other host or tumour models, we believe that our findings support the administration of gemcitabine at 11 HALO then CDDP at 15 HALO. Similar circadian patterns have been shown for the chronopharmacology of anticancer drugs across different rodent species or strains (Granda and Lévi, 2002). Mechanisms include rhythms in reduced glutathione content in liver and other organs and host tolerability for platinum complexes in rats and mice (Bélanger *et al*, 1988; Li *et al*, 1997) and rhythms in BCL-2 expression in bone marrow and host tolerability for docetaxel in B6D2F_1_ and C3H/He mice (Tampellini *et al*, 1998; Granda *et al*, 2001, 2005). A similar circadian pattern in the anticancer efficacy of the same drug has also been shown in different experimental tumour models (Granda and Lévi, 2002). For instance, platinum complexes were most active against rat plasmacytoma or mouse osteosarcoma following dosing near the middle of the night (Sothern *et al*, 1989; Granda *et al*, 2002), while docetaxel was most active against mammary MA13/C or pancreatic P03 carcinomas following dosing at daytime in mice from different strains (Tampellini *et al*, 1998; Granda *et al*, 2001).

The optimal circadian times for the delivery of gemcitabine and CDDP correspond to awakening and mid-activity in cancer patients, two circadian stages that can be identified with rest-activity monitoring, using a wrist-worn watch for 3–7 days (Mormont *et al*, 2000).

Thus, the rest-activity rhythm is the most overt output of the circadian timing system in mammals (Hastings *et al*, 2003). Many other rhythmic biologic functions that are relevant for the chronopharmacology of anticancer agents display a similar phase relation with the rest-activity rhythm both in rodents and in humans. These observations have led to refer the optimal treatment times to the onset of the rest phase of the rest-activity cycle (Lévi, 2001). For instance, dosing oxaliplatin near 1600 and 5-fluorouracil near 0400 proved to be a better schedule than constant rate infusion of the same drugs in a phase III trial of this combination in patients with metastatic colorectal cancer (Lévi *et al*, 1997). Another clinical trial with this combination has further confirmed these times as being best as compared to seven other ones staggered along the 24-h time scale (Lévi *et al*, in preparation).

Nevertheless, the circadian pattern in rest-activity was altered in one-third of patients with metastatic cancer. Furthermore, the ‘strength’ of the circadian component of the rest-activity rhythm displayed both a close relation with quality of life and an independent prognostic value for survival (Mormont *et al*, 2000; Innominato *et al*, 2005; Garufi *et al*, 2005). While the relevance of this rhythm for the prediction of chronotherapy efficacy is currently being investigated, we feel that rest-activity monitoring should be performed prior to the delivery of gemcitabine–CDDP chronotherapy. Presumably, a 2.5 h duration should be preferential for gemcitabine infusion (Tempero *et al*, 2003).

Such clinical chronopharmacologic development of gemcitabine-platinum combination is warranted in view of both the limited efficacy of current treatment regimens in advanced lung cancer and the three-fold survival benefit achieved here by the optimal circadian schedule as compared to concurrent dosing of both agents in the experimental model.

## Figures and Tables

**Table 1:** Body weight loss and extent of neutropenia at nadir according to gemcitabine dosing time, whether the drug was given alone or combined with CDDP in healthy mice, irrespective of interval between drugs

		**Gemcitabine dosing time (HALO)**	
**Toxicity**	**Gemcitabine**	**11**	**23**
Body weight loss (%)	Single agent	1.9±0.7	6.4±2.4
	Combined with CDDP	6.8±1.0	7.9±0.8

Neutrophil count (cells mm^−3^)	Single agent	386±24	250±25
	Combined with CDDP	365±28	224±28
